# Health-related quality of life in patients with neovascular age-related macular degeneration: a prospective cohort study

**DOI:** 10.1186/s41687-024-00775-z

**Published:** 2024-08-12

**Authors:** Anna-Maria Kubin, Ida Korva-Gurung, Pasi Ohtonen, Nina Hautala

**Affiliations:** 1 Department of Ophthalmology, Research Unit of Clinical Medicine and Medical Research Center, University of Oulu, P.O.Box 21, Oulu, OYS 90029 Finland; 2 Oulu University Hospital, Oulu, Finland; 3 Research Service Unit, Oulu University Hospital and University of Oulu, Oulu, Finland; 4 The Research Unit of Surgery, Anesthesia and Intensive care, Oulu University Hospital and University of Oulu, Oulu, Finland

**Keywords:** Neovascular age-related macular degeneration, Quality of life, Health-related quality of life, Anti-VEGF treatment, Elderly

## Abstract

**Background:**

Neovascular age-related macular degeneration (nAMD) is a common cause of visual impairment and blindness in the elderly with globally increasing prevalence. Vascular endothelial growth factor inhibitor (anti-VEGF) treatment has improved visual prognosis of nAMD, but continuous treatment may cause anxiety and stress, although increase in visual acuity (VA) may also have positive effects on patients’ quality of life. The health care burden due to frequent treatment and monitoring is apparent, but the effect of anti-VEGF treatment on patients’ quality of life is not fully understood. We evaluated the overall impact of nAMD and its treatment on newly diagnosed patients’ health-related quality of life (HRQoL) in real-world setting.

**Methods:**

The present prospective cohort study included newly diagnosed nAMD patients treated with anti-VEGF injections at Oulu University Hospital during 2019–2020. Data included parameters from comprehensive ophthalmic examination and fundus imaging, age at diagnosis, sex, comorbidities, visual acuity, and frequency of anti-VEGF injections. HRQoL was assessed by 15D questionnaire at diagnosis, 6 months, and 12 months.

**Results:**

95 nAMD patients were included. They were 78 ± 8 years old, 56 (59%) were female, and 74 (78%) had more than one comorbidity. The patients received 8 ± 3 anti-VEGF-injections. Visual acuity (VA) improved from 56 ± 18 to 61 ± 24 Early treatment diabetic retinopathy study (ETDRS) letters in 12 months. VA improved > 5 ETDRS letters in 45 (47%), remained stable in 30 (32%) and decreased > 5 letters in 17 (18%) eyes. The mean total 15D score reflecting overall HRQoL decreased from 0.850 ± 0.104 to 0.834 ± 0.103 in 12 months. Decreased HRQoL was associated with baseline best-corrected VA (BCVA) ≥ 70 ETDRS letters (*p* = 0.023) and more than one comorbidity (*p* = 0.034). HRQoL regarding visual function increased from 0.765 ± 0.194 to 0.789 ± 0.184 during the 12-month follow-up.

**Conclusions:**

In real world setting, HRQoL regarding visual function improved in anti-VEGF-treated nAMD patients during the first 12 months after the diagnosis and treatment initiation. Good baseline VA or several comorbidities were associated with decreased overall HRQoL during the follow-up. Despite the effectiveness of anti-VEGF treatment on visual function, several other aspects affecting elderly patients’ everyday life should be considered when nAMD treatment is implemented.

## Background

Age-related macular degeneration (AMD) is one of the leading causes of irreversible visual impairment and blindness in the elderly in high-income countries [1]. The pathogenesis of AMD is related to the ageing of the retina, the retinal pigment epithelium and Bruch’s membrane leading to development of drusen, retinal pigment epithelium (RPE) abnormalities, and macular neovascularisation within the choroid or the retina due to activation of angiogenesis by several molecules, such as vascular endothelial growth factor (VEGF) [2, 3]. Advanced AMD may cause decrease in visual acuity, loss of central vision, metamorphopsia, or changes in color vision [3]. Age is the most significant single risk factor for AMD. The proportion of inhabitants exceeding 65 years of age is growing in developed countries, which highlights the role of AMD as an important public health challenge. Neovascular AMD (nAMD) has a globally established treatment, which consists of regular ophthalmological follow-ups and monthly to bimonthly treatment with intravitreal injections of drugs inhibiting VEGF [4]. The anti-VEGF treatment has revolutionized the visual prognosis of patients with nAMD and the prevalence of visual impairment due to AMD has decreased in Finland since 2012 [5]. The health care burden is mostly affected by nAMD due to the continuous monitoring and frequent administration of anti-VEGF drugs. The incidence of nAMD has more than doubled during the past 15 years in older population in Finland [6]. In agreement, the prevalence of any AMD is expected to grow globally from 196 million in 2020 to 288 million in 2040 [7].

Eye diseases, including AMD, are associated with decline in visual acuity (VA), mental health and health-related quality of life (HRQoL) [8–11]. Visual impairment and declining VA secondary to nAMD may cause difficulties in performing every-day tasks such as reading and driving a vehicle, as well as increase the risk for accidents and injurious falls [9, 10, 12–14]. As a result, a majority of nAMD patients experience the need for assistance in every-day life indicating increased dependence on others [15].

Positive effects to reading abilities and psychological well-being have been noted due to anti-VEGF treatment induced increase in VA [15–17]. However, patients have reported anxiety and stress regarding to frequent and continuous intravitreal injections and follow-up. The impact of nAMD that may potentially have an effect on visual function and burden of its treatment on patient’s overall quality of life in real-life setting is not fully established. For this reason, we evaluated the impact of nAMD and its treatment by anti-VEGF agents on patients’ overall HRQoL during the first year after diagnosis. We expect that measuring HRQoL by using the 15D instrument will reflect well on treatment outcome as results from 15D HRQoL questionnaire takes into consideration the stress of the frequent treatment and the changes in independence and performing daily activities as well.

## Methods

### Study population and demographic data

A prospective cohort study was conducted at the Oulu University Hospital, which is responsible for the tertiary care of approximately 410 000 inhabitants in Finland. The study cohort consisted of 95 consecutive nAMD patients who had received diagnosis between 1.11.2019-31.10.2020. Inclusion criteria was newly diagnosed nAMD and initiation of anti-VEGF treatment. The diagnosis of nAMD was performed by an ophthalmologist utilizing comprehensive clinical examination, evaluation of best-corrected visual acuity (BCVA), and fundus imaging (fundus photography, fluorescein angiogram, optical coherence tomography (OCT), and optical coherence tomography angiography (OCT-A)) based on the discretion of the treating physician. The treatment and follow-up were performed according to the Current Care Guidelines for AMD and in the assessment of the treating physician [4]. Bevacizumab is the most common first-line choice for the initiation of anti-VEGF treatment in Finland. In the current study cohort, the intravitreal anti-VEGF treatment was initiated by four monthly injections of bevacizumab, and the treatment was discontinued if the macula was dry at control visit evaluated by OCT imaging. If intra- or subretinal fluid remained in central macular area, the treatment was continued by additional injections with 4–6 weeks intervals. If no sufficient treatment response was achieved by bevacizumab, another anti-VEGF agent (aflibercept) was used.

The demographic and clinical data of the patients was collected using Oulu University Hospital’s electronic data base and included parameters for age, gender, age at diagnosis of nAMD, comorbidities, VA, and number and frequency of anti-VEGF injections. The study eye was the eye first diagnosed with nAMD, or the eye with better VA in bilateral cases. Changes in VA were determined by using ETDRS letters [18].

### Health-related quality of life assessment

The study participants filled out a self-administered 15D questionnaire at the time of the diagnosis, 6 months, and 12 months after the onset of nAMD. The 15D is a generic and standardised instrument used to measure HRQoL in 15 dimensions of life: mobility, vision, hearing, breathing, sleeping, eating, speech (communication), usual activities, mental function, discomfort and symptoms, depression, distress, vitality, and sexual activity [19]. Each question contains five answer options on a scale of 1 (no difficulties) to 5 (extreme difficulties). A single 15D index score on a scale between 0 (representing HRQoL equal to being dead) and 1 (representing the best possible HRQoL) was obtained by weighting the scores with population-based preference weights [19]. The minimum important change (MIC) in 15D index score is change ≥ 0.015, which can be regarded as the threshold for improvement or deterioration of HRQoL [20]. In this study, we categorized favourable response in HRQoL if the 15D index score remained the same (change < 0.015) or improved (≥ 0.015). The preference weights of the 15D were obtained from several representative samples of Finnish adult population through a 3-stage valuation procedure and are used in an additive aggregation formula to generate the 15D score (single index number vH) over all the dimensions [19]. The rank correlations between averaged sets of importance weights from different population samples in 15D have varied between 0.94 and 0.96, and Pearson correlations between 0.97 and 0.98. Also, regression tests (regressing one averaged set on another) have indicated that the agreement is quite good [21].

### Statistical analysis

The summary measurements are presented as mean with standard deviation. The categorical data was analysed using *Χ*^2^- test or Fisher’s exact test, and Student’s t-test or Welch test was used for continuous data. We found a significant impact of good baseline visus of the better eye (≥ 70 ETDRS) on the change of QoL in univariate analysis, and to further explore this we performed four adjusted logistic regression models, which were adjusted with the worse eye visus and in addition with (1) age (< 75 vs. ≥ 75), (2) sex, (3) number of diseases (0–1 vs. > 1), and number of anti-VEGF injections (≤ 4 vs. > 4). Two tailed p-values are presented. SPSS (IBM Corp. Released 2021. IBM SPSS Statistics for Windows, Version 28.0. Armonk, NY: IBM Corp) was used for analyses.

### Ethical aspects of the study

The study followed the tenets of the Declaration of Helsinki, and the study was conducted with the approval of the Oulu University Hospital Research Committee (permission ID 221/2016). All patients were informed of the study protocol and a written consent of participation was obtained from each patient. Complete anonymity was adhered, and all study information is anonymized. The article does not include any data that may identify the person.

## Results

### Characteristics of the study population

The study population consisted of 95 newly diagnosed nAMD patients, out of which 56 (59%) were female. The mean age of the patients was 78 ± 8 years and 67 (71%) were ≥ 75 years old. More than one comorbidity was found in 74 (78%) patients. The characteristics of the study population are presented in Table 1. There were 8 (8%) bilateral nAMD cases at baseline. Eyes with nAMD received 8 ± 3 (range 1–13) anti-VEGF injections on average during the 12-month follow-up. One third of the patients received less than 4 injections in 12 months, whereas most patients had continuous injections throughout the first treatment year (Table 2). Bevacizumab was the first-line choice for the initiation of anti-VEGF treatment in all patients in the current cohort. 10 of the patients (11%) had later switch to aflibercept due to inefficient treatment response by bevacizumab.

An average baseline VA at the time of the diagnosis of nAMD was 56 ± 19 ETDRS letters and 61 ± 24 ETDRS letters after 12-month follow-up in the study eye. 79 (83%) patients had VA ≥ 70 ETDRS both at the baseline and at the end of follow-up. Six patients fulfilled the criteria of visual impairment (VA < 59 ETDRS in the better eye) at the baseline and 5 after 12-month follow-up. During the follow-up VA improved ≥ 5 ETDRS in 45 (47%) patients and remained the same in 30 (32%) patients. VA decreased ≥ 5 ETDRS in 17 (18%) patients.

**Table 1:** Characteristics of the study population

Characteristic	Total *n* = 95
Females, n (%)	56 (59)
Age at the diagnosis, years, mean (SD)	78 (8)
Age distribution	
< 75 years, n (%)	28 (30)
≥ 75 years, n (%)	67 (71)
Total comorbidities	
0–1, n (%)	21 (22)
>1 (%), n	74 (78)
Visual acuity, mean ETDRS^a^ (SD)	
Baseline	56 (19)
At 12 months	61 (24)
VA ≥ 70 letters^b^	
Baseline, n (%)	79 (83)
At 12 months, n (%)	79 (83)
Change in VA in ETDRS^a^ letters at 12 months	
Loss > 5 ETDRS letters, n (%)	17 (18)
No change and gain, n (%)	75 (79)

*SD* = standard deviation; *VA* = visual acuity

^a^The VA of the study eye was used

^b^The VA of the better-seeing eye was used

**Table 2:** The impact of demographic and treatment data on changes in quality of life during 12-month follow-up

Variable	Decrease in QoL, *n* = 39	Favourable response^a^ in QoL, *n* = 41	*p*-value^c^
Age ≥ 75 years, n (%)	28 (72)	28 (68)	*p* = 0.733
Males, n (%)	16 (41)	18 (44)	*p* = 0.795
Total comorbidities > 1, n (%)	35 (90)	29 (71)	*p* = 0.034*
VA ≥ 70 ETDRS^b^, n (%)			
Baseline	37 (95)	30 (77)	*p* = 0.023*
At 12 months, n (%)	34 (87)	33 (80)	*p* = 0.417
VA < 59 ETDRS^b^ (visual impairment), n (%)			
Baseline	1 (3)	5 (13)	*p* = 0.201
At 12 months	1 (3)	4 (10)	*p* = 0.360
VA loss > 5 ETDRS letters n (%)	6 (15)	7 (18)	*p* = 0.761
Number of anti-VEGF injections > 4, n (%)	27 (69)	28 (68)	*p* = 0.928

*VA* = visual acuity

^a^15D index score improved ≥ 0.015 or remained the same (change and < 0.015)

^b^The VA of the better-seeing eye was used

^c^Χ2- test or Fisher’s exact test

*Statistically significant *p*-value

### Changes in different 15D dimensions during follow-up

**Fig. 1:**
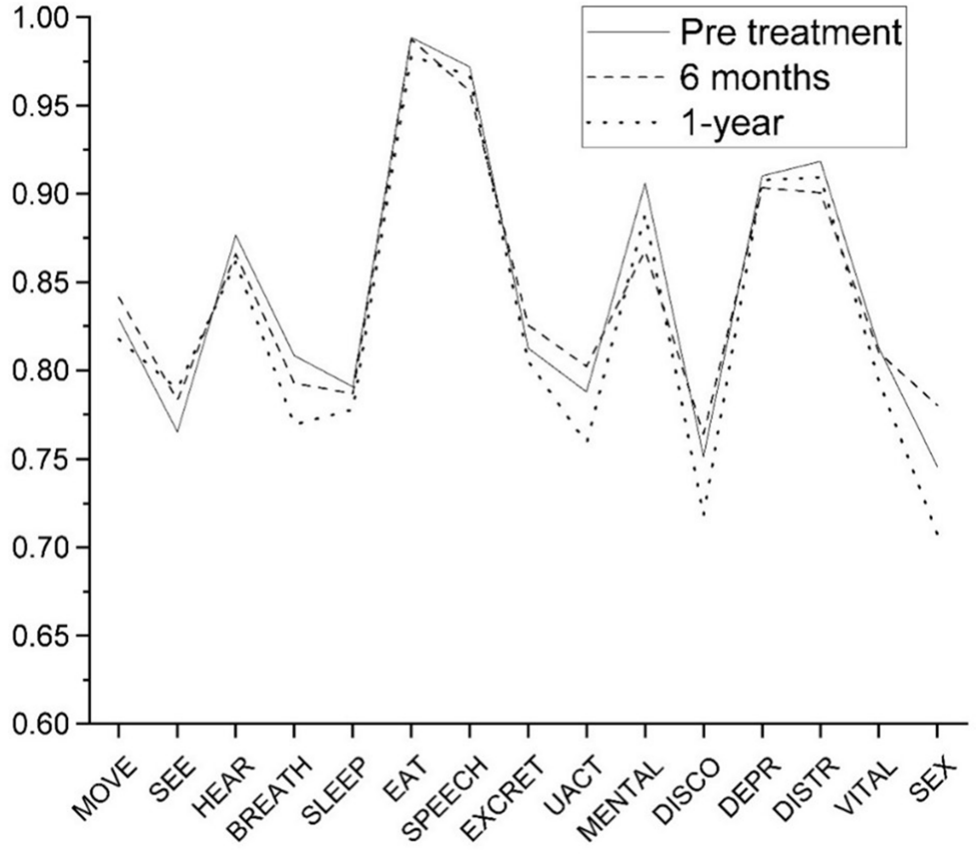
The 15D outcomes of the health-related quality of life (HRQoL) measurements in patients with nAMD at baseline, 6 months and 12-months follow up. *MOVE* mobility; *SEE* vision; *HEAR* hearing; *BREATH* breathing; *SLEEP* sleeping; *EAT* eating; *SPEECH* speech; *EXCRET* excretion; *UACT* usual activities; *MENTAL* mental functions; *DISCO* discomfort and symptoms; *DEPR* depression; *DISTR* distress; *VITAL* vitality; *SEX* sexual activity

Figure 1 shows the average 15D profiles at baseline, 6 months, and 12 months after nAMD diagnosis. When assessing individual 15D profiles, the vision dimension improved from 0.765 ± 0.194 to 0.789 ± 0.184 during the 12-month follow-up. The dimension indicative of performing usual activities in every-day life increased from baseline 0.788 ± 0.232 to 0.802 ± 0.213 at 6-month follow-up and decreased to 0.759 ± 0.225 at 12 months. Similar trend with initial improvement during the first 6 months was also documented in patients’ perceptions of “movement” dimension which first increased from 0.829 ± 0.196 at baseline to 0.841 ± 0.177 and then decreased to 0.818 ± 0.194.

### 15D index score representing overall health related quality of life

The mean 15D index score representing the overall HRQoL was 0.850 ± 0.104 at baseline and declined to 0.834 ± 0.103 during 12-month follow-up (*n* = 80). To further assess the impact of patient variables with changes in overall HRQoL, favourable response in HRQoL life was categorized when the index score improved ≥ 0.015 (MIC) or remained the same (change < 0.015). The 15D score improved in 24 (30%) patients and remained the same in 17 (21%) patients reflecting favourable response among study population (*n* = 41, 51%). The total 15D score decreased ≥ 0.015 in 39 (49%) patients reflecting poorer HRQoL at the end of follow-up. Associations between change in total 15D score and patients’ variables is shown in Table 2. Of the patients with decreased HRQoL 37 (95%) had VA ≥ 70 ETDRS in the better eye, whereas 30 (77%) patients with favourable outcome in the 15D score had VA ≥ 70 ETDRS. In the multivariable adjusted logistic regression models, the odds ratios for decrease in quality of life varied between 6.01 and 7.25 (p-value < 0.05 in all) (Table 3). The decreased HRQoL was also independently associated with multimorbidity as 35 (90%) patients with poor outcome in HRQoL had more than one comorbidity (*p* = 0.034). Other variables, such as age, sex or changes in VA, showed no statistically significant association with the patient reported in HRQoL.

**Table 3:** The impact of good baseline VA on decrease in QoL. Logistic regression models are adjusted with the worse eye visus and in addition with (1) age (< 75 vs. ≥ 75), (2) sex, (3) number of diseases (0–1 vs. > 1), and number of anti-VEGF injections (≤ 4 vs. > 4) in models 1–4, respectively

	Odds ratio	95% confidence interval	*p*-value
Model 1	6.20	1.22 to 31.6	0.028
Model 2	6.38	1.24 to 32.7	0.026
Model 3	7.25	1.40 to 37.6	0.018
Model 4	6.01	1.19 to 30.4	0.030

## Discussion

The current prospective study investigated the effect of anti-VEGF treated nAMD on HRQoL in the first 12 months after the diagnosis and initiation of treatment in real world setting. Vision related quality of life (VRQoL) improved with major increase during the first six months measured by the 15D. However, the overall HRQoL reflecting general health and satisfaction decreased during the first year after diagnosis and treatment initiation This is supported by the recent studies reporting the association of multimorbidity and lower HRQoL in the older population [22, 23].

Typically, nAMD patients experience an initial improvement in VA following initiation of intravitreal anti-VEGF-treatment before plateau [24]. In our study, VA improved or remained stable in a majority of patients (80%) uring the first 12 months after anti-VEGF treatment initiation which likely reflects in the improved VRQoL (improved 15D vision related dimension) yet further research is required to determine the stability of the treatment’s impact during longer follow-up. Previously there has been some variation in results when QoL of anti-VEGF treated nAMD patients has been studied with different QoL assessments. A Norwegian study found a significant increase in VRQoL during 12 months of anti-VEGF treatment on previously treatment naive nAMD patients when the better seeing eye was treated, however in contrast VRQoL was unaltered when worse seeing eye was treated despite significant improvement in VA [25]. In addition, a previous study demonstrated the improvement in VRQoL at 6 and 12 months of aflibercept-treatment, and there was a positive correlation between the increase in VA and VRQoL. However, in the study the mean number injections used was low (4.6) and VRQoL declined in patients who had received ≥ 9 injections [26]. In the present study patients received 8 ± 3 anti-VEGF injections on average during the 12-month follow-up.

Previously VA has been shown to have correlation with specific 15D dimensions including usual activities and mobility [8, 21, 27]. Our results show a transient increase in 15D dimension indicative of performing usual activities in every-day life as the score initially improved during the first six months but then declined at 12 months. Similar trend with initial improvement during the first 6 months was also documented in patients’ perceptions of “movement” dimension. One may assume that initiation of anti-VEGF treatment for nAMD with associated improvement in VA and healing of disturbing visual symptoms may have influenced on patient-reported experience on daily activities at first months of the treatment. Later, the stress from frequency and continuation of the injections and possible plateau in VA may have affected the 12-month outcome.

The mean 15D index score representing the overall HRQoL declined during the 12-month follow-up from 0.850 ± 0.104 to 0.834 ± 0.103, and overall 49% of the study patients experienced poorer HRQoL at the end of follow-up. Decreased total 15D score at 12 months was independently associated with baseline VA ≥ 70 ETDRS letters in the better eye (*p* = 0.023). VA of 70 ETDRS can be defined as relatively good or adequate VA as it is e.g. a requirement for standard driving license in the countries of the European union (directive 2006/126/EC) and thus affects individual’s daily living and its activities. On the other hand, VA below 70 ETDRS is considered as potential threshold for progressive decrease in HRQoL [28, 29]. The decrease in HRQoL might be influenced by slight deterioration in VA during the follow-up, as is characteristic for the disease, which may lead to the decreased ability for independent mobility, for example due to possible loss of driver’s licence. In addition, mild vision impairment (69–59 ETDRS) can also challenge performance of self-care [28] and affect negatively on HRQoL. On the other hand, if the baseline VA is good and remains stable the positive effect of treatment may have limited impact on overall HRQoL and the burden from the disease and treatment may affect the QoL.

The study patients with lower baseline VA might be more used to the restrictions of declined vision, may not have a driving licence to lose, and changes in vision due to nAMD may not affect their HRQoL as clearly as in those with better baseline VA. Gomi et al. showed an improvement in VRQoL on patients with BCVA over 70 ETDRS at baseline if the patient had experienced VA increase due to treatment [26]. However, 15D instrument used in our study takes into consideration other health-related dimensions of HRQoL, such as independence as well as the visual component which may partly explain the difference in these findings. Mobility has been linked to better HRQoL, and 3 dimensions of necessary mobility for the elderly have been demonstrated: utilitarian needs (accessibility), affective needs (independence or control) and aesthetic needs (viewing nature for example) [231].

Multimorbidity is defined by having ≥ 2 chronic diseases [31]. In our study, multimorbidity correlated with decreased HRQoL in an older population with nAMD (*p* = 0.034). In agreement, a meta-analysis including 39 articles concluded that multimorbidity had a negative effect on HRQoL, and especially the physical dimension of HRQoL compared to the mental component [31]. The average number of diseases of a single person increases in correlation with advancing age [32, 33]. The average age of the study patients was 78 ± 8 years and 78% of the patients had multiple comorbidities at the time of the diagnosis, which may at least partly explain the decrease in overall HRQoL at 12-month follow-up. Also, the common multimorbidity of the current study cohort consisting of older population with nAMD may cause additional difficulties for independent mobility and thus affect the HRQoL. Treatment adherence is critical in successful outcomes and improved visual prognosis in nAMD patients [34]. A recent meta-analysis showed that treatment dissatisfaction, multimorbidity or old age, and the burden of the continuous nAMD treatment are causes for discontinuing the treatment or being non-adherent [35]. Thus, there are multiple factors affecting the treatment outcomes and HRQoL in older population with nAMD, including the growing number of comorbidities, changes in independence and other psychosocial factors. Our results highlight the cumulating disease burden in the aging population and the need to examine general health factors in addition to the visual function when considering the effects of nAMD treatment on patient’s HRQoL.

Our study has some limitations including the relatively small number of participants (*n* = 95), which caused certain restrictions for achieving statistical significance in several notable changes in percentages in different variables. Prospective nature of the study and a real-life setting can be considered as strengths. The present study included only newly diagnosed treatment naive patients, who may experience the greatest positive impact on VA and VRQoL from the anti-VEGF treatment. The baseline HRQoL measurements from the treatment naïve nAMD patients may, however, reflect well the effect of nAMD related sudden onset of visual symptoms or anxiety of visual loss in addition to the further effects of anti-VEGF treatment, which supports the composition of the current cohort of newly diagnosed nAMD patients. The follow-up time of the study was limited to 12 months and noted positive impact on VA and VRQoL may diminish in time due to the natural course of nAMD. Therefore, future studies with longer follow-up and longitudinal settings are needed to demonstrate the long-term HRQoL results in older population with nAMD.

Neovascular AMD presents a challenge for health care resource planning as well as for the nAMD patients with declining visual function and HRQoL over time. New longer-acting treatment options for nAMD may decrease the economical and humane burden of frequent and continuous intravitreal anti-VEGF injections [36–38]. Further solutions, such as improvement of earlier diagnosis of nAMD, potential preventive factors of the disease, and more effective treatment options, are needed for the growing public health care challenge of increasing number of nAMD patients. However, our results indicated that current anti-VEGF treatment for nAMD led to stability or improvement of VA in most of the patients and improved VRQoL measured by 15D instrument. Treatment of nAMD might have had positive impact on the level of overall HRQoL scores despite the decline in total 15D score during the follow-up taken the multimorbidity, old age of the participants, and other related features affecting the HRQoL into consideration.

## Conclusions

nAMD is a common cause of visual impairment and blindness in the elderly with globally increasing prevalence. Anti-VEGF treatment has improved visual prognosis of nAMD. Increase in VA may have positive effects on patients’ QoL, but continuous treatment may cause burden. In real-world setting, overall HRQoL decreased, whereas QoL regarding visual function improved in older population with nAMD during the first 12 months after the diagnosis and initiation of anti-VEGF treatment. Good baseline visual acuity or several comorbidities were associated with decreased overall HRQoL during the follow-up. Despite the effectiveness of anti-VEGF treatment on visual function, several other aspects affecting elderly patients’ everyday life should be considered when nAMD treatment is implemented.

## Data Availability

The datasets used and/or analysed during the current study are available from the corresponding author on reasonable request.

## Abbreviations

AMD: Age Related macular degeneration
Anti-VEGF: Vascular endothelial growth factor inhibitor
BCVA: Best corrected visual acuity
ETDRS: Early treatment diabetic retinopathy study
HRQoL: Health-related quality of life
MIC: Minimum important change
nAMD: Neovascular age-related macular degeneration
OCT: Optical coherence tomography
OCT-A: Optical coherence tomography angiography
QoL: Quality of life
VA: Visual acuity
VEGF: Vascular endothelial growth factor
VRQoL: Vision related quality of life
